# Analysis of Tug of War Competition: A Narrative Complete Review

**DOI:** 10.3390/ijerph19010003

**Published:** 2021-12-21

**Authors:** Ruth Cayero, Valentín Rocandio, Asier Zubillaga, Ignacio Refoyo, Julio Calleja-González, Arkaitz Castañeda-Babarro, Inmaculada Martínez de Aldama

**Affiliations:** 1 Department of Physical Education and Sports, Faculty of Education and Sport, University of the Basque Country, (UPV/EHU), 01007 Vitoria-Gasteiz, Spain; ruth.cayero@ehu.eus (R.C.); valentin.rocandio@ehu.eus (V.R.); asier.zubillaga@ehu.eus (A.Z.); julio.calleja@ehu.eus (J.C.-G.); inmaculada.martinezdealdama@ehu.es (I.M.d.A.); 2 AFIPE Research Group, Technical University of Madrid, 28040 Madrid, Spain; 3 Health, Physical Activity and Sports Science Laboratory, Department of Physical Activity and Sports, Faculty of Psychology and Education, University of Deusto, 48007 Bilbao, Spain; arkaitz.castaneda@deusto.es

**Keywords:** tug of war, anthropometrics, physical capacities, physiology, injuries, kinetics

## Abstract

Tug-of-war (TOW) is an internationally played activity including professional and amateur athletes, defined as early as 4000 years ago (as a rope-less version) in the artwork on Egyptian tomb engravings, and is played as per the rules laid out by TWIF, which has 73 member countries and administrative headquarters in the USA. Typically, two teams of “pullers” participate and apply enormous contra directional forces on the pulling rope. Originally, two types of competition are used: knockout and points. This narrative review describes the scientific state of the art of TOW. To the best of the authors’ knowledge, no previous information has been published on this topic. Anthropometric parameters for competitors are near 83.6, lean body mass 69.4, and body fat 16. The VO_2MAX_ is 55.8 mL/kg/min. In terms of relative strength, the dynamic leg power is 4659.8 N. Endurance TOW elicits minimal muscle damage. Injured strains and sprains comprised over half of all injuries: back (42%), shoulder–upper limb (23%) and knee (17%). Pulling movement in TOW contests can be divided into three phases, namely the “drop”, “hold” and “drive” phases. The maximal pulling force was 1041.6 ± 123.9 N. The percentage of dynamic pulling force in the static maximal pulling force was 75.5 ± 14.4% and the dynamic ranged from 106.4 to 182.5%. There are two gripping styles: indoor and outdoor. The friction characteristics between surface and shoe in TOW is important in determining a suitable shoe for indoor TOW. A waist belt might be a useful piece of equipment for TOW sport. The EMG technique in TOW entails a high degree of dorsal muscle activity during the pulling. The factor of force vanishing was the coordination among athletes. The force vanishing percentage goes from 8.82 ± 5.59 for two contenders to 19.74 ± 2.22 for eight athletes, 6.4% in the sum of two pullers. However, in the drop phase, for female elite TOW team, only the 0.5% of the pulling force was wasted. Future studies are need in order to understand better this historical sport activity.

## 1. Introduction

Tug-of-war (TOW) is an internationally played activity and includes professional and amateur sport athletes [1,2]. TOW is one of the oldest sports in current existence, and Egyptian tomb engravings depict boys participating in this sport over 4000 years ago [1] with a long tradition, dating back to approximately 2000 BC. The term originates from the German “togga werra” which denotes “a contest in tugging or pulling” [3]. In this way, TOW was recorded as a royal sport in several ancient civilizations, such as China, Egypt and Greece. In particular, in ancient China, TOW was usually called “*hook pulling*” and its history can be traced back to the Spring and Autumn Period, i.e., more than 2500 years prior.

There is no specific time and place to pinpoint the origin of TOW. The contest of pulling a rope has also been described in histories from countries, such as India, China, Korea, France, Scandinavia, Great Britain, and South America. Towards the end of the 19th century, TOW became an organized sport in parts of Europe and it was also included in the Olympic Games held through 1900 to 1920 [3].

In recent decades, TOW has increasingly gained popularity on both national and international levels. The first association was created in England in 1958, and the TOW International Federation (TWIF), which controls global practice of the competition, was founded in the early 1960s. The TWIF is a member of the World Games Association, and the sport of TOW has been part of the World Games since 1981, when it was included for the first time in a World Games held in USA [2]. More recently, the sport has become organized on a worldwide basis. The TOW International Federation (TWIF) has 73 members in its association and has organized the international TOW competition since 1964 [3,4].

After describe a brief history about TOW, which has many decades of existence as a historical sport, we consider it to be of interest to analyze—from a scientific point of view—what is the current knowledge about this traditional sport. Therefore, this complete narrative review aims to describe all of the scientific state of the art of TOW, given that, to the best of the authors’ knowledge, no previous information has been published on this topic.

## 2. Methods

### 2.1. Information Sources

A computer-based scientific literature search was finished for the years 1900–2021 (6 November) by means of the following information sources: Medline (PubMed), Web of Science, the Cochrane Collaboration Database, Cochrane Library, Evidence Database (PEDro), Evidence Based Medicine (EBM) Search review, National Guidelines, EMBASE, Scopus and Google Scholar. The searches used the keywords: “TUG OF WAR SPORT”. Furthermore, this narrative review was conducted in accordance with the Preferred Reporting Items for Review Statement [5].

### 2.2. Study Inclusion Criteria

The researchers (R.-C., A.-K., I.-M.A., I.R.) obtained the titles and abstracts of all publications and determined the relevance of the publications for inclusion. The criteria for allocations of the articles were satisfied. The full text of each manuscript was obtained to ascertain if the publication satisfied the inclusion criteria. In addition, the reference sections of the selected articles were searched to identify other relevant articles. Finally, for the current narrative review, only studies focusing on TOW performance related to the following parameters were included: anthropometrics, physical and physiological capacities, injuries and kinematics analysis.

The following inclusion criteria were applied to each study: the data on the study source (including the authors and the year of publication), the study design, the sample size, and the characteristics of the participants (level, race and sex). Abstracts, non- peer reviewed papers and book chapters were excluded.

Finally, two authors (V.R. and J.C.-G.) independently extracted the final results of the interventions using a spreadsheet (Microsoft Inc., Seattle, WA, USA). Subsequently, disagreements were resolved by discussion until a consensus or third-party adjudication (A.-Z) was reached.

### 2.3. Study Exclusion Criteria

Other articles were not considered and duplicated articles were deleted. Articles regarding other team sports populations were not considered. The study also excluded articles related to patents that describe machines or devices to TOW practices. Finally, we excluded the articles on TOW that related to different areas of medicine

## 3. Competition

TOW is played as per the rules laid out by TWIF administrative headquarters in the USA. Typically, two teams of “pullers” participate and apply enormous contra directional forces on the pulling rope [2]. In this type of competition, there are two teams of eight, pulling against one another on a rope of not less than 33.5 m. The main target is to pull the opposing team towards a centerline for a distance of 4 m. Originally, two types of competition were used: knockout and points [3].

Teams are categorized based on body mass classes ranging from 480 kg to 720 kg per team, and also as men, women or seniors (>18 years old for males and >16 years old for females), U-23, and junior (15–18 years old) teams [6]. During the championships, the following body mass categories apply: male: 560-600-640-680-700 kg and female: 480-500-520-540-560 kg. On the other hand, there is another category (started in 2012) which is the mixed category, with 580 kg senior, 560 kg, and the U-23 and 520 kg junior [4]. The teams can make a change in all competitions, but the person who enters must weight equal to or less than the person which is being replaced [4].

If, in each body mass category, there are more than 11 teams, sub-groups are made. In these groups, each team throws twice against each opponent; one on one side and another, on the other side, can win or lose both or tie. In contrast, in the semi-finals there is no tie. If this happens, a third pull is made. The same happens in the finals and in the fight for the bronze medal [4].

Furthermore, the competitions are based on body mass categories. The individual athlete must “make weight” and the use of different body mass reduction strategies leading to acute dehydration is common [3]. The best of three pulls is the typical format [3], with a rest period of 2 min between the pulls. A maximum of 6 min may be claimed between trials, beginning when a team leaves the arena and ending when a team is in the marshalling area ready to re-enter to the arena [4].

## 4. Performance Characteristics

Descriptive Analysis is described in Table 1.

### 4.1. Anthropometrics

The international scientific literature describes the general characteristics of TOW athletes compared with a control group in only one descriptive and transversal study, to the best of the authors knowledge [3]. In this sense, some interesting data about general anthropometrical parameters have been described recently. For example, body mass 83.6 (3.0) kg; lean body mass (69.4 (2.1) kg; body fat: 16.7 (0.9) %; fat mass 14.2 kg; and height 1.81 cm.

### 4.2. Physical and Physiological Capacities

Related to the conditional capacities, in terms of endurance performance, these types of athletes presented 55.8 (1.6) mL/kg/min in the VO_2max_, measured by a treadmill test [3]. Therefore, aerobic power was higher in the TOW group than age and sex described parameters for the general population, but were below the values reported for elite endurance athletes [7].

Based on the scientific articles about strength, during either dynamic or static pulling in a TOW competition, the maximal pulling force on the rope might be higher than 150% of the participants’ body mass [8]. Regarding the relative strength, dynamic leg power was 4659.8 ± 151.6 N. In this way, given that strength is a key attribute of TOW, high levels of grip, back, and leg strength are essential to resist the large forces generated by the opposing team [3]. In this sense, previous studies presented a significantly higher strength to body mass ratio when adjusting for body mass [3]. In particular, international level TOW males have excellent strength and above average endurance, relative to their body size [2]. On the other side, a group of researchers investigated gender differences in pulling strength during experimentally executed TOW for Japanese elementary school children [9]. This study concluded that pulling strength gender difference was large in the fifth and sixth grade. However, no tendency was found from first to fourth grade. In addition, pulling strength tended to grow constantly. Regarding female children, the sum of pulling strength increased substantially when the grade changed from second to third and from fourth to sixth, and the sum of back strength and rope tension were very close to each other. The results suggested that, although male children experience more muscle growth, female children obtain better motor functioning than males [9]. Finally, another interesting study observed that, after three weeks of specific training, there resulted significant neuromuscular changes, increasing muscular strength and strength endurance [10].

Concerning flexibility, little information was found. Leg flexibility was 25.4 (2.0) cm for the TOW group [3]. The sit and reach (goal standard) and forward flexion tests were performed as a measure of lower back and hamstring flexibility and back flexion test, as a measure of the flexibility of the back extensor muscles, presenting nearly 25.4 cm (2.0) in the sit and reach test, 8.0 cm (2.1) in forward flexion and 28.6 cm (1.4) in back flexion test [3]. The mean values for forward flexion (8 (2) cm) and back flexion (28.6 (1.4) cm) for the TOW group were below the expected ranges that is used as standards for athletes (10–25 cm and 35–50 cm, respectively).

From a holistic point of view, Rider and colleges examined the physiological effects of training and competition with 17–18 male pullers [11]. Each participant’s fitness was assessed (flexibility, power, muscular strength, and body composition) at two moments in the training period (pre-training) and (post-training). The pullers’ mean hydration levels decreased during training, but hydration status returned to baseline levels 56 h after the event. Elevated CK levels appear to reflect the intense nature of this practice. The data concluded that endurance TOW elicits minimal muscle damage compared with pre-training, similar to other endurance activities in terms of its physiological impact on the body [11].

## 5. Injuries

Trainers, athletes and researchers are concerned about injuries that may arise from exhausted muscle use caused by the tremendous forces imposed on the rope during TOW games [12]. Given this, pullers participate and apply enormous contra directional forces on the pulling rope [3], related to the injuries, strains and sprains that have been described. Over half of all injuries were comprised of injuries affecting the back (42%), shoulder–upper limb (23%), and knee (17%). In both genders, injury patterns were similar [1]. Finally, in a previous survey study of 252 elite outdoor TOW pullers, 35% of them reported one or more previous injuries during TOW training or practice [1]. Injuries in the UE (Upper extremity) and shoulder girdle accounted for 12% and 11% of the injured areas; the UE and shoulder girdle are reported as the third and fourth most frequently injured areas in the same study [1].

In addition, there are several reports of medical complications, such as hernias [12], retinal hemorrhage [13] and fractures [4,14]. Finally, in addition, one study published described a tear in a bicep muscle [15].

## 6. Kinetics Analysis

The description of pulling technique is important for understanding this particular sport. The pulling position and the relationship between the body inclination, holding height and pulling force has been studied [15,16]. The elite TOW pullers produce the motion to pull by more than their arms; the entire body is used as well. This is achieved by holding the arm close to the side of the body, extending the trunk and inclining the body and lower body heavily [16]. Analyzing the synchronize pulling timing and direction using a drone, all pullers employ a synchronized rightward and backward movement, resulting in the synchronized pulling timing and direction, which culminates in lowering the loss of force [17]. In this way, as suggested by a group directed by Tanaka, there is great importance on skill in terms of timing the drop phase to avoid the loss of team pulling force in TOW, concluding that the smaller the peak time differences in two pullers, the smaller the reduction of peak force in the pair [18].

The pulling movement in a TOW contest can be divided into three phases, namely the “drop”, “hold” and “drive” phase: (1) the drop phase is the stage during which pullers rapidly employ a pulling force right after they start pulling; (2) the hold phase is the stage during which the pullers hold against the pulling of their opponent [16,19]; and (3) the drive phase, which is described as an exerting pulling force with backwards walking which draws the opponent into their own territory [8]. The elite team’s backward pulling distance of the drop phase is longer than a normal team and the anchor man pulls the rope shorter than other positions, comparatively, given that they have a different role [19].

Concretely analyzing the drive phase of elite indoor TOW athletes, the maximal pulling forces was 1041.6 ± 123.9 N, and this ranged from 792.3 to 1240.7 N. The percentage of dynamic pulling force in the static maximal pulling force was 75.5 ± 14.4% [8]. The dynamic pulling forces expressed as a percentage of body mass ranged from 106.4 to 182.5%. They also described the order of lower leg and lower limb movement [8].

Following on from the team’s pulling force, the team’s resulting force is smaller than the sum of individual players [20]. The factor of force vanishing was the coordination among athletes. The force vanishing percentage is from 8.82 ± 5.59% for two contenders, to 19.74 ± 2.22% for eight athletes, being 6.4% of the sum of two pullers [18]. However, in the drop phase, for a female elite TOW team, only 0.5% of the pulling force was wasted [21].

Concreted kinetic analysis provided information on the use of each UE muscle group during TOW movements [22]. In particular, this information regarded the individual power hold (148.5 ± 31.7 kg) and the team power hold (1188 kg). In this sense, the gripping force, the force fluctuation and the force pulse variables were analyzed [23]. The results showed that the TOW athletes exhibited superior force-generating capacity and more economic force-grading as compared with the non-athletes, without additional costs to task accuracy and force steadiness, during a highly-demanding rhythmic force task.

In other ways, other studies investigated the influence of specific types of training programs. The first of these analyzed the influence of specific types of muscle training, performed by previously well-trained competitive athletes on the force-velocity of the arm flexors. In particular, four rowers, five athletes competing in TOW and six middle- and long-distance runners were measured at different stages of their training program over the period of one training year [24]. The main conclusion is that variation in the type, intensity, and volume of arm training throughout a year hardly affected the course of the force-velocity curve of the arm flexors of well-trained athletes [24]. The second research study analyzed the plyometric strength training effects of elite male tuggers, related to Defend Fast Break (DFB) and Attack Fast Break (AFB), before and after practice [25]. The parameters of DFB, which were the time of the peak pull force, the minimal pull force, the action response time and the average amount of force before and after the training, presented at significant levels. In the AFB parameter, the first peak and minimal pull force, the time of the first peak and the time of action responses, the average of force and the slope of force, were deemed to be at a significant level. There are some previous studies about the pulling force curves in DFB and AFB [26]. They suggested to take the DFB movements in order to produce powerful pulling force, and to then transform the AFB movements to maintain the team formation.

Concerning pulling stiles, two are described in the literature [27]: the “European Back-Step” (EBS) and the “Japanese Back-Step” (JBS). These are both attack movements used in indoor TOW. The kinetics parameters of these two different attacks were found in order to conclude which movement style was more powerful and more efficient. The JBS was more powerful than EBS, both in the peak and the minimum GRF.

To the best of our knowledge, only one study has compared the ground reaction force between the gripping styles. In this study it was found that the same pullers exhibit a higher ground force when using indoor gripping (GS1) than when using outdoor gripping (GS2) [28], given that the GS1 group may have a better chance of winning in real competitions as the force level and the speed to achieve the peak force are both beneficial for dominating sports competitions [29]. Moreover, the more flexed elbow and more supinated forearm observed in the GS2 group might be better for the main strong elbow flexor (m. biceps brachial) in order to generate its maximal contraction in an anatomical and tension length aspect [30]. A more supinated forearm was found to reduce the gripping force [31] and might have a negative impact on force performance in the GS1 group.

Based on joint movements, the lower activation in the proximal elbow muscles might be due to their poor joint position and muscle length for the generation of maximal contraction [30] during TOW pulling, i.e., muscles are in a lengthening position for flexing movements and shortening position for extending movements. Although a higher co-contraction ratio (CCR) might help in providing joint stability and preventing injuries to the peri-joint soft tissues during competition, previous studies suggested that higher joint stiffness from muscle co-contraction can result in higher mechanical and physiological stress on joint cartilages [32]. Avoidance of long-lasting UE training along with closed chain or weight bearing exercises to reduce the excessive mechanical force [33] and sufficient resting between training activities might be helpful.

Frictional forces act in the contact plane of two bodies and, thus, one of them being the earth’s surface, horizontal forces can be generated. In this sense, the weight is an important factor that affects friction; the greater the weight and the maximum static friction force is, the greater the advantage [34]. The friction characteristics between the surface and shoe in TOW were investigated. In this study, the static coefficient of friction at each shoe on three different mats (English, European and Japanese) was measured in order to determine a suitable shoe for indoor TOW. When TOW is attempted on an English mat or a Japanese mat, the use of TOR 107 or TOR 109 shoe types might be much preferable to the tennis shoe or running shoe. However, there were no significant differences among all shoes on a European mat [35].

In relation to materials and in order to find the benefits of the waist belt (WB) in TOW, a study was conducted comparing the kinematic differences between two-handed (TH) and WB pulling backward exercises [36]. Since there was no significant difference between the mean maximal forces of TH and WB trials in the drive phase, it could be assumed that WB would produce a pulling force that is no less than that produced during a TH trial without using the upper limbs. In addition, the backward walking speed during WB trials was 1.5 times faster than that of TH. In addition, the mean speed of COM during the WB trials was 1.6 times faster than TH. These results suggest that the WB had the efficacy to accomplish a given task in the drive phase.

In relation to the EMG technique, this concept has been widely used in studying muscle activation in sports research [37]. In TOW, the EMG describes a high degree of dorsal muscle activity during the pulling [22].

Finally, other studies going into the detail of measuring systems and machines presented an experiment for constructing an it-TOW, finding that to put it-TOW into practice, the pulling force data must be exchanged and not be measured by a load cell, but instead defined by another system [38]. Analyzing TOW, under the conditions of the maximum friction and a stable rope, achieves a sequence which can exert maximum energy [39]. Later, Bing Zhang, in 2012, built an ideal and simple model with mechanical analys [40]. They studied the maximum pressure and conducted a force analysis of rope, concluding that if the sequence is arrayed from short to tall and only when the heights are the same, should the athletes with the greater weight stand behind. In addition, Chun-ta Lin et al. proposes a theoretical biomechanical measurement and analysis system to evaluate the performance and, consequently, verify the effectiveness of the training process [41].

## 7. Conclusions

In summary, TOW is an internationally played activity including professional and amateur sport athletes. TOW is typically a competition with two teams of “pullers” who participate in and apply enormous contra directional forces on the pulling rope. Originally, two types of competition are used: knockout and points. The anthropometric parameters are near 83.6 kg, lean body mass 69.4, and body fat 16%. The endurance values presented are VO_2max_ 55.8 mL/kg/min. Regarding the relative strength, the dynamic leg power was 4659.8 N. Endurance TOW elicits minimal muscle damage. Injured strains and sprains comprised over half of all injuries, and the back (42%), shoulder–upper limb (23%), and knee (17%) were most common sites of injury. Some medical complications, such as hernias, retinal hemorrhage and fractures, have been described as well. The pulling movement in TOW contests can be divided into three phases: namely the “drop”, “hold” and “drive” phases. The maximal pulling force was 1041.6 ± 123.9 N. The percentage of dynamic pulling force in terms of static maximal pulling force was 75.5 ± 14.4% and the dynamic pulling forces expressed as a percentage of weight ranged from 106.4 to 182.5%. There are two gripping styles: indoor and outdoor. The friction characteristics between surface and shoe in TOW mean that it is important to determine a suitable shoe for indoor TOW. A waist belt might be a useful piece of equipment for TOW. The EMG technique in TOW is described as a high degree of dorsal muscle activity during the pulling. The factor of the force vanishing was the coordination among athletes. The force vanishing percentage goes from 8.82 ± 5.59% for two contenders, to 19.74 ± 2.22% for eight athletes, and it 6.4% of the sum of two pullers. However, in the drop phase, for the female elite TOW team, only 0.5% of the pulling force was wasted.

### 7.1. Practical Applications

Our narrative review provides an important first approach toward the progress of knowledge performance for TOW athletes. This first approach can be useful for practitioners in order to improve the performance in this particular traditional sport.

### 7.2. Future Lines

This particular sport needs more research in order to understand better the holistic performance.

## Figures and Tables

**Table 1:** Studies published related to Tug of War.

Anthropometrics						
Title	Authors	Year	N	Method	Variables	Results
Physiological and metabolic characteristics of elite tug of war athletes	Warrington, G; Ryan, C; Murray, F; Duffy, P; Kirwan, J. P	2001	16 male 34 ± 2 years	Collected data were comparing with a group of rugby forwards from the international squad	Anthropometrics	body mass: 83.6 (3.0) kg; lean body mass (69.4 (2.1) kg body fat: (16.7 (0.9) %) fat mass 14.2 kg height 1.81 cm
**Physical and Phisiologycal Capacities**						
Effects of 3-weeks intense training on physiological capacities of tug-of-war team	Northuis, M. E. and Cook, B	1998	9 males 6 males (control)	3-week training period Pre period Post period	Muscular strength Strength endurance Power Body composition Lower back and hamstring flexibility Body water volume Blood lactate	3-week training resulted ns. Neuromuscular changes ↑ muscular strength and ↑ strength endurance. ↑ lactate clearance time indicate sig. systemic metabolic changes.
Physiological and metabolic characteristics of elite tug of war athletes	Warrington et al.	2001	16 male 34 ± 2 years	Collected data were comparing with a group of rugby forwards from the international squad	VO_2MAX_ Strength Muscular power Flexibility Biochemical profile	BM: TOW< RF VO_2MAX_: TOW < RF Relative VO_2MAX_: TOW > RF Max HR: TOW = RF Bf: TOW < RF Composite strength: TOW > RF CMJ: TOW < RF Leg flexibility: TOW > RF Bag flexibility: Erythrocyte volume: TOW < RF
The Strain of The Pull: Examining the Physiological Effects of An Endurance Tug of War	Rider et al.	2017	15 male	3 weeks to prepare. Pre-Train and Post Train test. Blood and urine were collected at four time points (PreTrain, PostTrain, PullDay, and PostPull)	Flexibility Power Muscular strength Body composition Blood Urine SG CK.	Flexibility ↑ (24.42 ± 5.2 vs. 31.03 ± 6.1 cm, *p* < 0.05) CK: Pre-Train (2113.7 ± 1207.6) > Post Train (598 ± 73) Pull Day (1384.8 ± 936.6) > Post Pull (910.7 ± 244.7) Hydration levels ↓ during training (PreTrain:1.02 ± 0.01 vs. PostTrain: 1.03 ± 0.01 vs. PullDay: 1.03 ± 0.01
**Injuries**						
Tug-of-War Injuries: A Case Report and Review of the Literature	Pranit, N. C. and Abdelgawad, A. A.	2002	1 10 years man	Case Report		Measures should be taken to increase the awareness about these safety rules and prevention of consequent injuries
Tug of war: introduction to the sport and an epidemiological injury study among elite pullers	Smith, J. and Krabak, B	2002	252 31 ± 9.5 years 187 males 65 females	Survey during World TOW Championships in 1998	Demographic data Participation history Injury history during TOW Injury number Injury type	Strains: >50% Sprains: 42% Shoulder–upper limb: 23% Knee: 17% Similar between males and females.
Trauma resulting from tug-of war	Ferguson, A. and Kierkegaard, E.	1981	1	Case Report	No data has been found	No data has been found
Adult bochdalek hernia after playing at a tug of war	Liai et al.	1997	1 38 years Female	Case Report	No data has been found	Hernia repair with direct suturing through a thoracotomy
Tug-of-war hand: transforearm amputation by an unusual mechanism	Bruce W. And Hayes C.W.	1999	1 21 years man	Case Report	No data has been found	Amputation below elbow Rehabilitation Prosthesis
Extensive retinal hemorrhage after a game of tug-of-war	Moran M.	1984	1	Case Report	No data has been found	Extensive retinal hemorrhage
Injuries During a Massive Tug-of-War Game	Pei-Hsin Lin et al.	2003	1 64 years man	Case Report	No data has been found	Comprised liver and spleen rupture with C5-6 spinal cord Bilateral brachial plexus injuries
Arm Pain from Tug of War	Khosravi et al.	2006	1 16 years man	Case Report	No data has been found	Tear of the biceps muscle
**Kinetics Analisys**						
Influence of Training on the Force-Velocity Relationship of the Arm Flexors of Active Sportsmen	de Koning et al.	1984	15 National level: 4 rowers (20–28 years) 6 runners (17–36 years) 5 athlete’s TOWS (27–42 years)	1 training year 3 measured stages	FVC Max. mechanical power Torques Angular velocities	Force-velocity characteristics of muscle of previously well-trained sportsmen can hardly be influenced
Biomechanical analysis on tug of war	Yamamoto et al.	1988	16	Hold session during 10 s	Body mass Grip strength Back strength Power hold Power stroke EMG	EMG: high activity of dorsal muscle Ind. power hold 148.5 + −31.7 kg Calculated team power hold: 1188 kg. Real team power hold: 792 kg
Influences of some sports shoes on the strength of pulling exercise in Indoor Tug-of-War	Yamamoto, et al.	1997	8 males 28.1± 2.95 years 176.3 ± 4.65 cm 77.6 ± 5.39 kg	4 types of shoes 3 different mats Maximal pull on each shoe for 5 sg	Static coefficient of friction PF at each shoe	English or Japanese mat: TOR 107/TOR 109 European mat: ns differences
Biomechanical considerations of pulling force in tug of war with computer simulation	Kawahara et al.	2001	1 21 years active college student.	Biomechanical model of human body: 5-segmented 3 joints	CG SCG Height Body mass Holding height	Holding height vs. PF pulling force. sig. correlation Body inclination vs. PF sig. correlation
A three-dimensional motion analysis of two-handed and waist belt pulling backward exercises in elite tug of war athletes	Tanaka et al.	2004	20 Males 28.3 ± 3.3 years 174.4 ± 4.3 cm 71.9 ± 6.0 kg	Each subject performed TH and WB pull in the DF at his maximal effort	Static maximal pulling forces Stride length Stride frequency Walking speed	TH vs. WB sig. correlation The speeds of backward walking: 0.2 ms^−1^ in TH 0.3 ms^−1^ in WB The stride lengths: 0.2 m in TH and WB Stride frequency: 1.4 steps/s TH 1.6 steps/s WB
Dynamical Analysis of Indoor Eight People Make Tug of War Attack Movements—’European Back-Step’ and ‘Japanese Back-Step’	Fong-Wei Wang and Chien-Lu Tsai	2005	8 22.1 ± 2.4 years 174.1 ± 3.6 cm 72.7 ± 2.4 kg	The 3D data EBS & JBS attack movements were analyzed	Peak a minimum of GRF	Peak backward GRF: JBS (1.9 bw) > EBS (1.85 bw). Minimum backward GRF: JBS (1.55 bw) > EBS (1.47 b w)
The analysis of pulling force curves in tug-of-war	Jui Hung Tu et al.	2005	11 Female 17.8 ± 0.99 years 163.9 ± 2.98 cm 59.1 ± 4.21 kg	3 trials of pulling force curves in DFB and AFB movement The rest time: more than 10 min	MaxF MinF AveF RT FS	MaxF, MinF, and FS sig. different in DFB and AFB Time-related parameters ns.
The study of team resultant force vanishing percentage in elite tug of war players	ChunHui Liou et al.	2005	9 Female 16.9 ± 0.6 years 163.8 ± 2.7 cm 58.7 ± 4.3 kg senior	6 kinds of team pulling: (A) two players (B) three players (C) four players (D) five players; (E) six (F) seven players (G) eight players	Sum of individual maximal PF (kqf) Team maximal PF Force vanishing %	The sum of maximal individual PF > team maximal PF. More number of players ↑ vanishing %
Characteristics of pulling movement for Japanese elite tug of war athletes	Nakagawa et al.	2005	16 2 teams Elite team Average team.	2 cameras recorded 2 trials. 2D motion analysis system was used	Analysis points on body: 8 points 6 angles	Produced the motion to pull by arm and body. To hold arm to body: closed their side, trunk, inclined their body and lower body Inclined their upper body slightly in comparison with average team
Biomechanical Analysis on dynamic pulling skill in elite indoor tug of war athletes	Tanaka et al.	2005	20 male	Each subject performed TH pull in the DF at his maximal effort	Maximal PF Dynamic PF Static PF Anatomical landmarks of the body	Maximal PF: 1041.6 ± 123.9 N Maximal PF: 201.8 ± 38.2% relative BM Dynamic PF: 149.0 ± 23.1% divided by the weight
Analysis of timing skill of drop exercise in elite indoor tug of war athletes	Tanaka et al.	2006	30 male 22 World Indoor TOW 2004 Champions 8 novice male students	Load cells with a strain amplifier connected to a pen oscillograph, in paper speed of 25 mm/sg A strobe light synchronized with a pen oscillograph	PT PT exerted by two pullers. Individual PeF PeF exerted by two pullers	The sum of individual PeF in two pullers was 305.9 ± 41.4 kgw and PeF exerted by the two pullers was 286.3 ± 38.8 kgw, 93.6% of the sum PeF in skilled pullers. 6% loss of PeF in skilled pair. Smaller PT differences are in two pullers The smaller is the reduction of PeF in pair
Fundamental experiment for constructing it-tow bio	Nakagawa et al.	2006	1 A healthy female participant no experience with TW 22 years 162 cm 529.2 N	PF measured in 3 tests and 3 trials per one test: -Drive phase -Hold phase	PF	PF data must be exchanged and not be measured by a load cell
Backward pulling distance in drop phase for Japanese elite Tug-of-war athletes	Nagahama et al.	2007	80. 5 elite teams (finalists) and 5 normal teams (non finalists) Women Lightweight division	Pulling distance on 1 sg of DF The BDP distance on DF was measured	BPD	Elite team pulled the rope longer than normal team Anchorman pulled the rope shorter than other positions comparatively
The biomechanical analysis for plyometric strength training effect of elite male tuggers	Lin, J. D et al.	2007	11 male high school 16.8 ± 0.99 years 171.59 ± 3.18 cm 70.14 ± 2.65 kg	Plyometric strength training machine of TOW for 8 weeks Pre-training and post-training FC	DFB AFB PF AveF MaxT MinF RT/FS	DFB: MaxT, MinF, RT AveF sig. differences pre-post training AFB: the first peak MinF, Max] and RT, AveF, F] sig. differences
Team pulling technique of the Tug-of-war—A birds eye analysis of TOW	Mukwaya et al.	2007	10 teams matches were recorded in All Japan women’s Tug of War Championship	2D motion analysis system Angles 1st line 2nd line	PF Loss of force The slanting angles (Sum of 7 angles)	0.5% of team PF was wasted in first DF The lateral slanting has very little relation with the loss of the force in DF
A cross-sectional study of gender differences in pulling strength of tow for Japanese elementary school children	Sato et al.	2009	16 children 8 male 8 female	The participants performed 1 trial for each parameter. The pulling mini game (4vs4) was set for 30 s and performed 3~5 games for each pairing	Back strength PF	Difference between rope tension and sum of pulling strength in male > females
Parametric analysis in tug of war based on ideal biomechanical model	Bing Zhang	2012	No data has been found	Parametric analysis in TOW Ideal biomechanical model	Maximum pressure Conduct force analysis of rope	The sequence is arrayed from short to tall and only when the heights are the same the athletes with the greater weight should stand behind
The Origin Development and Winning Skills of Tug of War	Xinyu Li	2015	1 puller of each team	The force analysis	a center of gravity of body Static friction force on the ground Force pulls The size of hand grip	Weight is the important factors: The greater the weight and the maximum static friction force is Work time is one of the important factors to success
Team pulling technique of elite female indoor-tow athletes from a drone’s point of view	Nakagawa et al.	2016	16 2 women team	2 games were filmed the R side of the competition lane. 2D motion analysis system. Video camera at roof of gymnasium Analysis time was 10 s of drop phase	X, Y-axis for all puller Foot position.	Synchronized movement: rightward and backward, caused synchronize pulling timing and direction, which culminated in lower the loss of the force
The novel biomechanical measurement and analysis system for tug-of-war	Chun-ta Linl et al.	2016	No data has been found	Digitizing system for collecting body segment parameters	PF Force curve Joint Moment Trunk segment Thigh segment Leg segment	Theoretical methods for PF estimation and joint moment analysis modules have been derived Experimental verification for the proposed system is being carried out
Differences in Force Gradation between Tug-of-War Athletes and Non-Athletes during Rhythmic Force Tracking at High Exertion Levels	Yen-Ting Lin et al.	2016	32 16 elite males 21.5 ±0.6 years 177.3 ± 4 cm 82.1 ± 5.7 kg; 16 non athletes 21.3 ± 0.6 years 174.1 ± 3.3 cm 64.4 ± 7.7 kg	Isometric handgrip (3 times of 20 sg)	Grip force Force fluctuation Force pulse variables	TOW athletes exhibited: ↑ Fmean ↑ ratio Fmean to body mass, ↑superior force-generating capacity ↑ economic force-grading.
Contribution of upper limb muscles to two different gripping styles in elite indoor tug of war athletes	Wen-Tzu Tang et al.	2017	20 Athletes 1 group GS1 16.5 ± 0.7 years 172.0 ± 4.2 cm 68.0 ± 6.6 kg 2 group GS2 16.8 ± 0.3 years 172.4 ± 4.9 cm 69.2 ± 7.4 kg	Pulling on a tug machine, participants used GS1 or GS2 their own habitual gripping style to pull for 5 in 15 sg trials. 14-segment anthropometric Kwon3D system.	Max F Max T Avg T Min T Max T-Min T PT COG Body tilting posture Surface EMG signal of UE muscles	Force and kinematic measurements showed a significantly better force performance and higher centre-of-gravity tilting angle with the GS1 than with (GS2) Higher and more symmetrical muscle activation detected by normalized surface electromyography signal amplitude was found in the GS2 group In both groups, the distal and flexor muscles were more activated than the proximal and extensor muscles, respectively

Legend: ↑: increase/↓: decrease/AFB: Attack fast break/AveF: Average Force/BF: Body Fat/Bf: Back flexion/BPD: Backward pulling distance/CK: creatine kinase/CMJ: Counter movement jump/COG: Center of gravity/DFB: Defend fast break/EBS: European Back-Step/FS: Force Slope/FVC: force-velocity curve/FC: Force curve/GS1: Gripping style one/GS2: Gripping style two/GRF: Ground Reaction Force/JBS: Japanese Back-Step/Max HR: Maximal heart rate/MaxF: Maximal pulling Force/MaxT: Time of the peak pull force/MinF: Minimum pulling force/ns: No significant/PeF: Peak Force/PF: Pulling Force/PT: Peak force time/RF: Reference group/RT: Reaction Time/SG: specific gravity/sig: significant/TH: Two handed/TOW: Tug of war/VO_2MAX_: maximal oxygen uptake/WB: Waist belt/CG: Segment center of gravity/SCG: Synthesis center of gravity.

## Data Availability

No new data were created or analyzed in this study. Data sharing is not applicable to this article.
